# Novel Treatment Strategy for Patients With Urea Cycle Disorders: Pharmacological Chaperones Enhance Enzyme Stability and Activity in Patient‐Derived Liver Disease Models

**DOI:** 10.1002/jimd.70043

**Published:** 2025-05-27

**Authors:** Adhuresa Ramosaj, Mariia Borsuk, Jarl Underhaug, Déborah Mathis, Shirou Matsumoto, Adrian Keogh, Vanessa Banz, Amit V. Pandey, Nadine Gougeard, Vicente Rubio, Aurora Martinez, Gabriella Allegri, Martin Poms, Beat Thöny, Johannes Häberle, Alexander Laemmle

**Affiliations:** ^1^ Division of Pediatric Endocrinology, Diabetology and Metabolism, Department of Pediatrics, Inselspital, Bern University Hospital University of Bern Bern Switzerland; ^2^ University Institute of Clinical Chemistry, Inselspital, Bern University Hospital, University of Bern Bern Switzerland; ^3^ Department of Biomedicine University of Bergen Bergen Norway; ^4^ Department of Pediatrics Graduate School of Medical Sciences, Kumamoto University Kumamoto Japan; ^5^ Department of Visceral Surgery and Medicine Inselspital, Bern University Hospital, University of Bern Bern Switzerland; ^6^ Department of Biomedical Research University of Bern Bern Switzerland; ^7^ Instituto de Biomedicina de Valencia (IBV‐CSIC) and Group 739 of CIBER de Enfermedades Raras (CIBERER‐ISCIII) Valencia Spain; ^8^ Division of Clinical Chemistry and Biochemistry University Children's Hospital, University of Zurich Zurich Switzerland; ^9^ Division of Metabolism and Children's Research Center (CRC) University Children's Hospital, University of Zurich Zurich Switzerland

**Keywords:** human induced pluripotent stem cell‐derived hepatocytes, human induced pluripotent stem cells, liver disease model, ornithine transcarbamylase deficiency, pharmacological chaperones, urea cycle disorders

## Abstract

Urea cycle disorders (UCDs) are inherited diseases causing recurrent life‐threatening metabolic decompensations due to impaired hepatic ammonia detoxification and decreased ureagenesis. Ornithine transcarbamylase (OTC) deficiency (OTCD) is X‐linked and the most common and often fatal UCD. In male hemizygous patients, disease severity primarily depends on the pathogenic sequence variant, while in heterozygous females, disease severity also depends on the X‐chromosomal inactivation (XCI) pattern. Females with unfavorable XCI predominantly expressing the mutant OTC protein may be severely affected. Here, we investigated a novel treatment strategy for OTCD since there is an unmet need for better therapies. In the first step, we performed a high throughput screening (HTS) using a diversity library with 10 000 chemical compounds to identify pharmacological chaperone (PC) candidates that stabilize purified wild‐type OTC. Stratification of our HTS results revealed five potential PCs, which were selected for further experimentation in cellular systems using primary human hepatocytes (PHHs) and human induced pluripotent stem cell (hiPSC)‐derived hepatocytes (hiPSC‐Heps) from healthy controls and OTCD patients. Two PCs—PC1 and PC4—increased OTC protein stability and activity in control hiPSC‐Heps, while PC4 in addition increased OTC activity in patient‐derived PHHs from a female OTCD patient with unfavorable XCI. Finally, PC1 and PC4 both significantly increased ureagenesis in patient‐derived PHHs. To conclude, we identified two PCs that stabilized wild‐type OTC and enhanced enzyme activity and ureagenesis. Our work suggests that PCs could provide a novel treatment strategy for OTCD specifically in females with unfavorable XCI.

**Abbreviations**
ALT: alanine aminotransferase
AST: aspartate aminotransferase
CPS1: carbamoyl phosphate synthetase 1
DSF: differential scanning fluorimetry
hiPSCs: human‐induced pluripotent stem cells
hiPSC‐Heps: hiPSC‐derived hepatocytes
HTS: high throughput screening
LDH: lactate dehydrogenase
OTC: ornithine transcarbamylase
OTCD: OTC deficiency
PAINS: pan‐assay interference compounds
PCs: pharmacological chaperones
PHHs: primary human hepatocytes
UCDs: urea cycle disorders
UCEs: urea cycle enzymes
XCI: X‐chromosomal inactivation

## 1 Introduction

Ornithine transcarbamylase (OTC; EC 2.1.3.3) deficiency (OTCD; OMIM #311250) is an inborn error of metabolism causing impaired ammonia detoxification and ureagenesis due to pathogenic variants in the *OTC* gene. OTCD is the most common urea cycle disorder (UCD) with reported incidences ranging from 1:70 000 to 1:14 000 [1, 2, 3, 4]. Patients diagnosed with OTCD often present with severe neurological symptoms due to hyperammonemia and remain at lifelong risk of recurrent and potentially life‐threatening episodes of metabolic decompensation [5, 6, 7, 8].

The *OTC* gene is located on the X chromosome. Males harboring a hemizygous pathogenic variant in the *OTC* gene are often severely affected, whereas in heterozygous females, disease severity also depends on the X‐chromosomal inactivation (XCI) pattern [9, 10]. Females with favorable XCI predominantly expressing the wild‐type OTC protein are considered healthy carriers, whereas females with unfavorable/skewed XCI expressing predominantly the mutant OTC protein may suffer from severe and fatal OTCD. Treatment strategies are based on a low‐protein diet, use of nitrogen/ammonium scavengers as well as l‐arginine and/or l‐citrulline supplementation [11, 12, 13, 14]. To date, the only curative approach is liver transplantation, which has limitations primarily due to donor shortage and invasiveness with potential short‐ and long‐term complications [15]. Other therapies are currently under investigation but are still not approved for clinical use as recently reviewed [16]. Therefore, novel and alternative treatment options are urgently needed [17].

One potential approach relies on the ability of certain chemical compounds—generally designated as pharmacological chaperones (PCs)—to interact with specific target proteins, hereby enhancing their stability [18, 19, 20] or lysosomal trafficking [21]. The first approved PC for an inborn error of metabolism is used for Fabry disease [22], while various compounds are currently being evaluated for other metabolic diseases [23, 24, 25, 26, 27, 28, 29]. Regarding the treatment of UCDs, *N*‐carbamyl‐l‐glutamate can be considered a chemical chaperone used for the treatment of some carbamoyl‐phosphate synthetase 1 (CPS1)‐deficient patients [30].

The majority of *OTC* variants are missense/nonsense changes causing misfolding of the OTC enzyme [31, 32]. In this work, we hypothesize that PCs could be used as a novel therapeutic strategy to either rescue the activity of the mutant OTC enzyme and/or enhance the residual enzyme activity in patients with reduced wild‐type OTC enzyme expression [19].

Here, we demonstrate that the selected compounds PC1 and PC4 increase wild‐type OTC protein expression and OTC enzymatic activity as well as ureagenesis in cellular models. These results suggest that PC1 and PC4 hold the potential to develop a novel PC‐based therapeutic strategy, specifically in heterozygous female patients with unfavorable/skewed XCI expressing only a little wild‐type OTC enzyme.

## 2 Materials and Methods

### 2.1 Study Approval

This study was approved by the local ethics committee (Canton of Bern, Switzerland; project ID: 2020‐02979) with prior written informed consent from all study subjects.

### 2.2 Preparation of Purified Human Wild‐Type OTC Protein

Human OTC (residues 33–354 of UniProt KB entry P00480; lacking the N‐terminal 32‐residue signal sequence cleaved upon mitochondrial entry) was expressed from a pET 22b (+) (Novagen) plasmid hosting the coding sequence for human OTC without its first 96 bases (pET 22‐human OTC). Expression of human OTC protein in *Escherichia coli* was performed as described previously [33]. It was purified from the postcentrifugal supernatant of the initial *E. coli* extract by a modification of the method of Marshall and Cohen for bovine OTC [34]. The pure enzyme was concentrated by ammonium sulfate (3.8 M) precipitation or by centrifugal ultrafiltration through a 30k membrane (Amicon Ultra15 device, from Merck). In the last case, the concentrated solution (about 8 mg/mL, determined by the Bradford commercial assay from Bio‐Rad with bovine serum albumin as standard) was brought to 20% glycerol by the addition of 87% glycerol. In both cases, the protein was aliquoted and stored at −80°C until use.

### 2.3 Differential Scanning Fluorimetry (DSF)‐Based High Throughput Screening (HTS)

The DSF‐based screening was performed using one batch of purified recombinant human OTC and the 10 000 compound library MyriaScreen Diversity Collection (TimTec/Sigma‐Aldrich) with 384‐well microtiter plates (Roche Applied Science). The final volume in each well was 25 μL, including 0.1 mg/mL wild‐type OTC (2.5 μM) in PBS buffer, pH 7.4, with 5× Sypro Orange dye (Sigma‐Aldrich, St. Louis, MI, USA), and 4% DMSO in controls (16 wells included DMSO controls on each plate) or 0.08 mg/mL compound (average, 250 μM based on the average molecular weight for the compounds in the whole library) and 4% DMSO. The thermal unfolding curves were registered by following the fluorescence of Sypro Orange (*λ*_ex_ = 465 nm and *λ*_em_ = 610 nm). The unfolding was measured from 20°C to 99°C at 2°C/min using a LightCycler 480 Real‐Time PCR System from Roche.

The experimental unfolding curves for OTC were normalized to reflect the fraction of unfolded protein, and the half‐denaturation temperature (*T*_m_) values were calculated as the maxima of the first derivative of the curves using the HTSDSF Explorer software [35]. For each plate, *T*_m_ values for OTC were obtained in the presence of each compound, while the average reference denaturation temperature (*T*_m,ref_) ± standard deviation (SD) was calculated using the 16 DMSO controls/plate. The Δ*T*_m_ values for OTC with each compound were defined as *T*_m_ − *T*_m,ref_. The cutoff value for hit selection was set to Δ*T*_m_ ± 1°C, which corresponds to 5× SD for a typical plate. Validation of compound hits was performed by concentration‐dependent DSF assays (Table S1) [35].

### 2.4 Cell Culture of Primary Human Hepatocytes (PHHs)

Control PHHs were isolated from resected liver tissue from a non‐affected (regarding OTCD) patient undergoing liver surgery. OTCD_4F PHHs were isolated from the patient's liver explant. We received prior informed consent from both patients. PHHs were plated on rat tail collagen type I‐coated plates at a density of 6.0 × 10^4^ cells/cm^2^ in Dulbecco's minimum essential medium supplemented with 10% fetal bovine serum, 2 mM GlutaMax (from ThermoFisher), 50 U/mL penicillin, 50 μg/mL streptomycin, and each 1 μM dexamethasone and insulin. After overnight culture, the medium was replaced by serum‐free Williams E Medium GlutaMax (no phenol red) containing the same concentrations of penicillin/streptomycin, dexamethasone, and insulin.

### 2.5 Testing of Potential Pharmacological Chaperones (PCs) in Cells

The five potential PCs (PC1–PC5) were purchased as follows: PC1, MolPort‐001‐013‐958, Vitas‐M Laboratory Ltd., catalog number STK156422; PC2, MolPort‐001‐521‐858, Vitas‐M Laboratory Ltd., catalog number STK735439; PC3, MolPort‐000‐512‐881, Vitas‐M Laboratory Ltd., catalog number STK805714; PC4, MolPort‐002‐093‐362, Specs, catalog number AG‐205/37386064; and PC5, MolPort‐004‐951‐942, TimTec, catalog number ST020780. The five potential PCs were tested in cells at 100 μM for 24 h. After washing cells with PBS, the same concentration of compounds was added with a concomitant ^15^NH_4_Cl challenge at 1 mM for the indicated time (usually 24 h). Urea secretion and ureagenesis were determined in the cell culture supernatant after ^15^NH_4_Cl challenge.

### 2.6 Assays to Determine Urea, Ureagenesis, AST, ALT, LDH, and Amino Acids

To assess urea secretion and cellular release of AST, ALT, and LDH, cell culture supernatants were collected at the indicated time points and centrifuged at 700 *g* for 5 min at 4°C. Urea was determined using the Quantichrom Urea Assay Kit (Bioassay Systems). Ureagenesis was determined as previously described [36]. AST, ALT, and LDH were determined by Roche Diagnostics Cobas 8000 analyzer to exclude cytotoxicity. Based on brightfield microscopy images and normal levels of liver enzymes in the cell culture supernatants, no toxic effects for PC1‐ and PC4‐treated cells were observed. Therefore, these data were not completely included in each consecutive experiment. Amino acids were analyzed by ion exchange chromatography with post‐column derivatization with ninhydrin reagent (Biochrom 30+ series).

### 2.7 Computational Modeling of the Interaction of PC1 and PC4 With OTC

Chemical structures of PC1 and PC4 were generated with OpenBabel software [37] from their SMILES format. PC1 and PC4 were then docked into the structure of wild‐type OTC protein (PDB 1OTH, crystal structure of human ornithine transcarbamoylase complexed with *N*‐phosphonacetyl‐l‐ornithine). Docking was performed with AutoDock VINA [38]. We performed 25 rounds of docking runs for each molecule, followed by clustering and rescoring the poses for binding after energy minimization.

### 2.8 Reprogramming of Fibroblasts Into hiPSCs and Differentiation of hiPSCs Into hiPSC‐Heps

One batch of fibroblasts was reprogrammed into hiPSCs and cultured as previously described [39]. hiPSCs were subsequently differentiated into hiPSCs‐Heps reproducing embryonic developmental stages as previously established [36]. Six to eleven different biological replicates of three independent hiPSC‐Hep differentiations were used to generate the respective figures.

### 2.9 Western Blot

Protein expression was analyzed by western blotting using a 10% SDS‐PAGE and antibodies targeted against human OTC, CPS1, and B‐Actin as described previously [36].

### 2.10 OTC Activity Assay

OTC activity in cell culture lysates (25–50 μg total protein) was determined based on previously described protocols [36, 40]. After adding ornithine and carbamoyl phosphate, and coloration with diacetylmonoxime/antipyrine/Fe, citrulline quantity was measured spectrophotometrically using the intensity of the absorption at 464 nm. Citrulline amount was considered proportional to the OTC enzyme activity.

### 2.11 Statistics

All experiments were repeated with at least three biological replicates, which were not stated otherwise. To compare groups, statistical analysis was performed using GraphPad Prism and Student's *t*‐test. For multiple group comparisons, ordinary one‐way ANOVA or Kruskal–Wallis tests were performed, as mentioned in figure legends. Data are expressed as mean ± standard error of mean (SEM).

## 3 Results

### 3.1 Identification of Five Compounds—Potential PCs—Which Stabilize OTC

**FIGURE 1:**
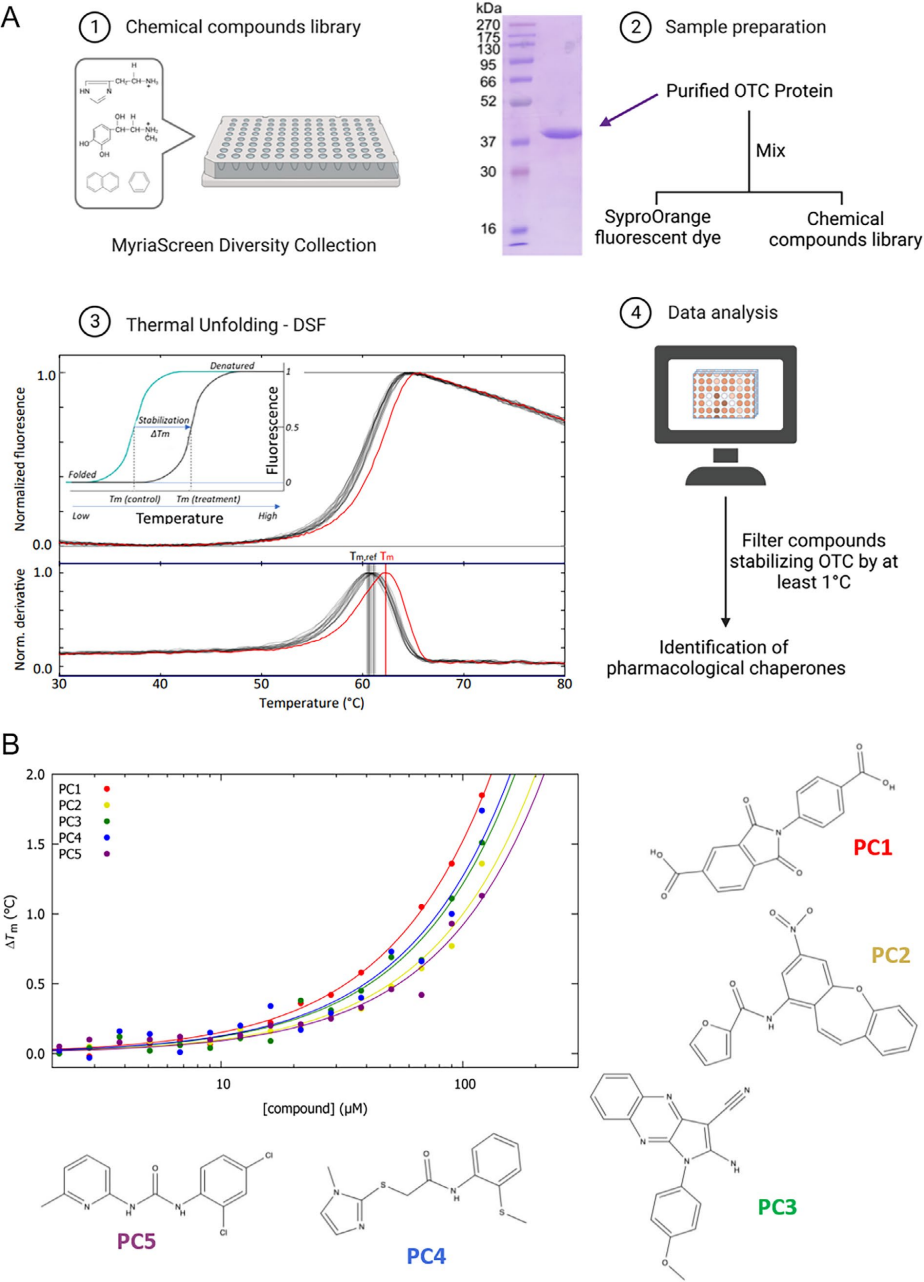
Identifying pharmacological chaperones (PCs) stabilizing human wild‐type OTC protein by high throughput screening. (A) Scheme of the steps for high throughput screening of stabilizers of OTC by differential scanning fluorimetry (DSF). (1) The compound library used was the MyriaScreen Diversity Collection consisting of more than 10 000 small molecule compounds. (2) The protein target for the screening was recombinant purified human wild‐type OTC, and the protein purity is illustrated by Coomassie‐stained SDS‐PAGE in a 12% polyacrylamide gel. Left lane, protein standards with defined molecular mass (kDa). (3) Recording of DSF‐monitored normalized unfolding curves (upper panel) and first derivatives (lower panel) for OTC in PBS buffer with DMSO (gray) and with one stabilizing hit compound in the same concentration of DMSO (PC1, red). *T*_m,ref_ (= 60.80°C ± 0.28°C; 16 curves) and *T*_m_ for PC1 = 62.28°C. Inset, schematic representation of the DSF method for determination of the thermal shifts, Δ*T*_m_. (4) Analysis of the data, calculation of the thermal shifts Δ*T*_m_ (= *T*_m_(with compound) − *T*_m,ref_(DMSO control)) and selection of stabilizing hits providing Δ*T*_m_ ≥ 1°C; performed with HTSDSF Explorer software. Figure created with BioRender.com. (B) The selected *primary hits* were validated through DSF assays evaluating the increase of Δ*T*_m_ values as a function of compound concentration. The figure shows the results for the selected *validated hits* with pharmacological chaperone potential for OTC (PC1–PC5), with their chemical structures. Figure created in part with BioRender.com.

To select stabilizing compounds with potential PC effect on OTC protein, HTS was performed by DSF‐monitored thermal unfolding with the MyriaScreen Diversity Collection Library (10 000 compounds; see Section 2 and Figure 1A; Panel 1) using purified human OTC (Figure 1A, Panel 2). The wild‐type form of human OTC was used in the screening, as disease‐associated variants show a tendency to aggregate and usually do not perform well in lengthy screening campaigns. The DSF‐monitored thermal unfolding of wild‐type OTC in PBS with 4% DMSO shows a single transition with a half‐denaturation temperature (*T*_m_) of 60.5°C ± 0.9°C, calculated from the average of all DMSO controls (Figure 1A, Panel 3; standard deviations for each plate were low, typically 0.2°C).

Data analysis provided the thermal shifts, Δ*T*_m_ (= *T*_m_(with compound) − *T*_m,ref_(DMSO control)), revealing 125 compounds that increased the *T*_m_ for OTC above the threshold of Δ*T*_m_ > 1°C (Figure 1A, Panel 4). After the elimination of hits for which visual inspection of the denaturation curves revealed artifacts, repetition of the DSF‐assays, and exclusion of hits corresponding to pan‐assay interference compounds (PAINS) [41], the number of primary hits was reduced to 50. These were further validated by DSF assays evaluating the increase of Δ*T*_m_ values as a function of compound concentration. Five validated hits (PC1–PC5) (Figure 1B) presented optimal concentration‐dependent stabilization of OTC, indicating ligand binding and protein stabilization [42]. These five compounds were selected for further in vitro testing, namely 2‐(4‐carboxylphenyl)‐1,3‐dioxo‐2,3‐dihydro‐1H‐isoindole‐5‐carboxylic acid (PC1), *N*‐{5‐nitro‐2‐oxatricyclo[9.4.0.0^3^,^8^]pentadeca‐1 (11),3,5,7,9,12,14‐heptaen‐7‐yl}furan‐2‐carboxamide (PC2), 2‐amino‐1‐(4‐methoxyphenyl)‐1H‐pyrrolo[2,3‐b]quinoxaline‐3‐carbonitrile (PC3), 2‐[(1‐methylimidazol‐2‐yl)sulfanyl]‐N‐[2‐(methylsulfanyl) phenyl]acetamide (PC4), and [(2,4‐dichlorophenyl) amino]‐N‐(6‐methyl(2‐pyridyl)) carboxamide (PC5).

### 3.2 Testing Selected Potential PCs in Functional Assays in PHHs

**FIGURE 2:**
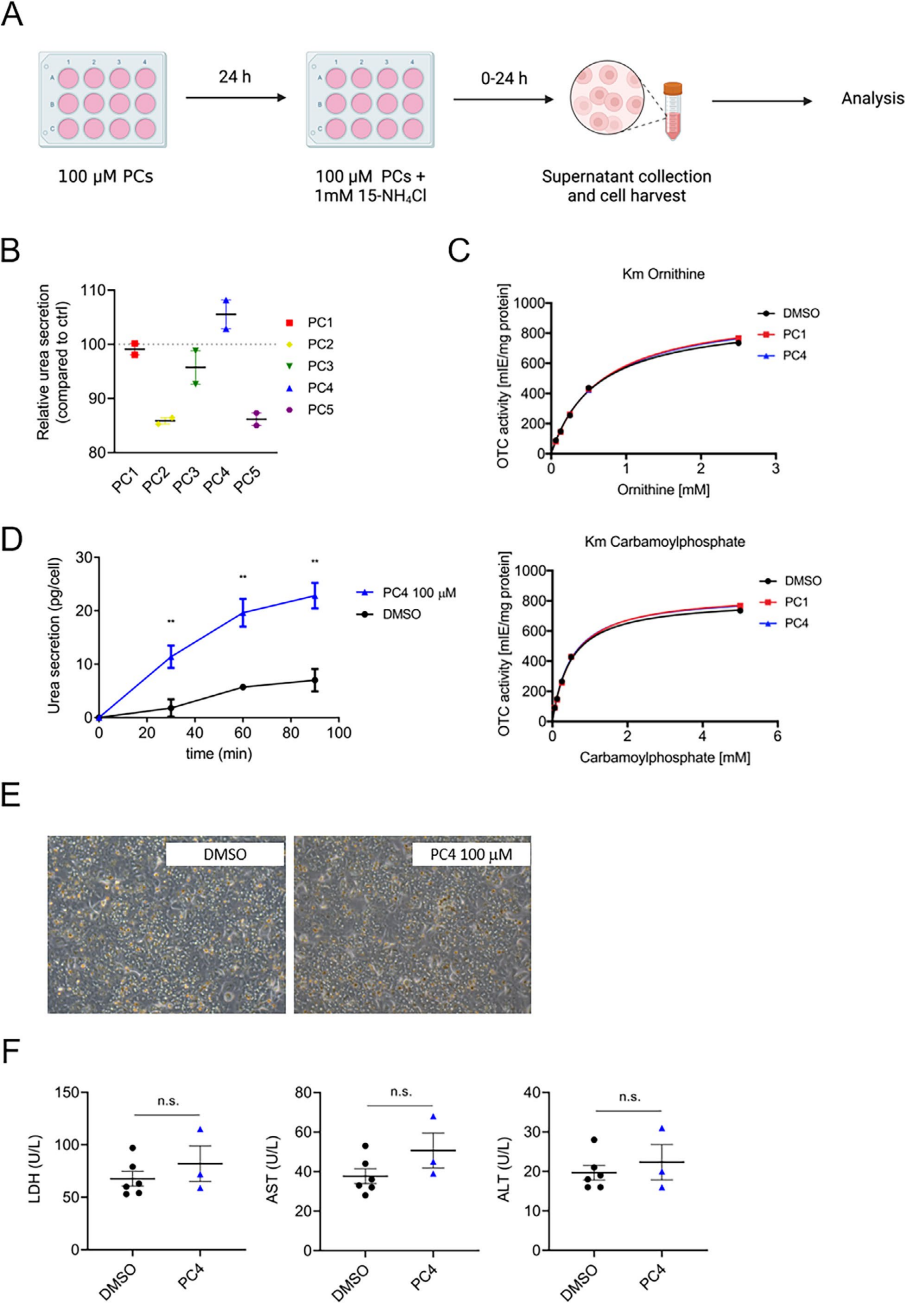
Testing of pharmacological chaperones (PCs) in primary human hepatocytes (PHHs). (A) Experimental scheme of testing PCs in PHHs. Cells were treated with 100 μM of PC dissolved in DMSO for 24 h, followed by a change of medium using the same PC concentration and concomitant challenge with 1 mM ^15^NH_4_Cl for additional 24 h—where not differently indicated—and consecutive determination of urea secretion and assessment of cytotoxicity. Controls were treated with similar amounts of DMSO. Created with BioRender.com. (B) Urea secretion in PHHs in response to exposure with PC1 to PC5 compared to DMSO‐treated control cells represented by the dashed line. (C) Substrate kinetics for ornithine and carbamoyl phosphate in cell lysates of PHHs incubated with 100 μM PC1 and PC4 compared to DMSO controls. (D) Urea secretion in PHHs in response to short‐term exposure (30, 60, and 90 min) with PC4 compared to DMSO‐treated control cells. (E and F) Brightfield microscopy imaging and assessment of LDH, AST, and ALT to exclude PC4‐related cytotoxicity. Data represent the average of two to six independent biological samples (B, D, and F) and single data points for the curves obtained in (C). Unpaired Student's *t*‐test. n.s., not significant. Error bars represent SEM.

Next, the five potential PCs (PC1–PC5) identified as OTC stabilizers selected from the HTS were tested in PHHs according to the scheme shown in Figure 2A. It is known from previous studies performing HTS that PCs often bind to the active site of their targets and hence may inhibit the enzymatic activity through competitive inhibition with their natural substrates [43]. In the case of OTC, the two substrates carbamoyl phosphate and ornithine are converted into citrulline. To assess or exclude PC‐mediated inhibitory effects, we first performed a straightforward functional and established assay in PHHs—we determined urea secretion in cell culture supernatants in response to an ammonia (^15^NH_4_Cl) challenge and concomitant PC treatment. We hypothesized that PCs that bind to OTC and inhibit its enzyme activity would result in reduced urea secretion. Therefore, PHHs were pretreated with 100 μM of PCs for 24 h, followed by a medium change using the same PC concentration and concomitant challenge with 1 mM ^15^NH_4_Cl for an additional 4 h prior to urea determination (Figure 2A,B). PC2, PC5, and to a lesser extent PC3 decreased urea secretion and hence were suspected to inhibit OTC enzyme activity (Figure 2B). Therefore, they were excluded from further testing. In contrast, PC1 and PC4 did not inhibit urea production (Figure 2B), and in line with this, none of these two compounds affected substrate kinetics when OTC activity was assayed in cell extracts (Figure 2C). Based on the results from the urea secretion assay, PC4 seemed to be the most effective compound in stabilizing OTC protein. Therefore, further experiments with PC4 were performed. For example, PHHs were pretreated with 100 μM of PC4 as described above (Figure 2A,B); however, shorter exposure periods to ^15^NH_4_Cl (30, 60, and 90 min) were chosen after the medium change and concomitant challenge with 1 mM ^15^NH_4_Cl (Figure 2D). PC4‐treated cells revealed significantly increased urea secretion compared to DMSO‐treated controls as early as 30 min after the medium change (Figure 2D). No cytotoxic effects were observed as assessed by brightfield microscopy (Figure 2E) and by determination of ALT, AST, and LDH (Figure 2F).

Taken together, these results suggest a specific interaction of PC4 with the OTC enzyme resulting in increased overall urea cycle activity as assessed by significantly enhanced hepatocellular urea secretion in response to a ^15^NH_4_Cl challenge.

### 3.3 Computational Modeling of the Interaction of PC1 and PC4 With OTC Protein

**FIGURE 3:**
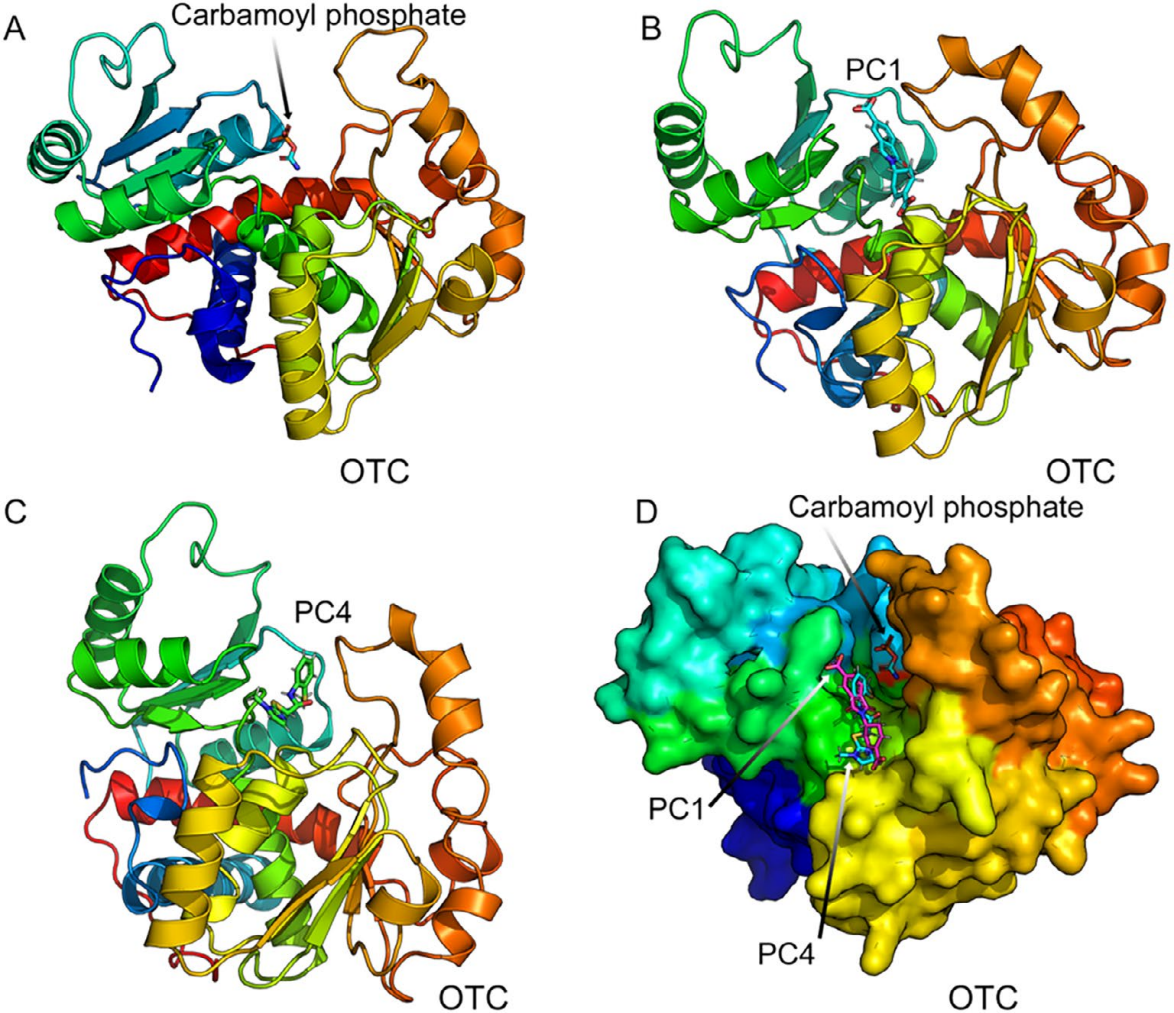
Computational modeling of the interaction of PC1 and PC4 with wild‐type OTC protein. Three‐dimensional structures of OTC subunit (from PDB 1FVO) in complex with carbamoyl phosphate (A), with PC1 (B) or PC4 (C). These two PCs were modeled under the guidance of in silico docking (see Section 2). (D) Surface view of the OTC subunit in complex with carbamoyl phosphate, PC1 (magenta), and PC4 (cyan). Structures of all three complexes are superimposed to show the different locations of their binding. Structures are colored in rainbow colors. Structures were generated using Pymol (www.pymol.org) and rendered as ray‐traced images using POV‐Ray (https://www.povray.org/).

In the next step, to assess the expected interactions of PC1 and PC4 with wild‐type OTC protein, we performed a computational modeling approach. Chemical models were generated with OpenBabel software from the SMILES of PC1 and PC4, which were then docked into the structure of wild‐type OTC subunit three‐dimensional structure (PDB: 1OTH) [44] obtained from the RCSB database (www.RCSB.org) (Figure 3A). Both PC1 (Figure 3B) and PC4 (Figure 3C) docked on the surface of the OTC subunit but did not seem to interfere with the formation of the OTC trimer or with substrate binding (Figure 3D) (checked with structures hosting the bisubstrate analog *N*‐phosphonoacetyl‐l‐ornithine or binding both carbamoylphosphate and with the ornithine analog l‐norvaline; respective Protein DataBank files 1OTH and 1C9Y; [45, 46]), supporting a protein stabilization role of both PC1 and PC4. Further analysis of the structural docking models for binding of PCs to OTC revealed interactions of both PC1 and PC4 with Aspartate 165, Asparagine 198, Histidine 202, and Serine 267 of the OTC subunit, indicating a binding hotspot of these chaperones close to the ornithine binding site (Table S2). Calculated binding energy and dissociation constant (*K*_D_) values for the best binding poses (Table S2) of the OTC subunit with PC1 were 6.6 kCal/mol and 13.7 μM, while for PC4, these values were 5.7 kCal/mol and 59.9 μM, respectively.

Taken together, these results suggest that PC1 and PC4 interact with wild‐type OTC protein and improve protein stabilization.

### 3.4 Modeling Ornithine Transcarbamylase Deficiency With Patient‐Derived hiPSC‐Heps

We recently established and published an in vitro liver disease model of OTCD with patient‐derived hiPSC‐Heps recapitulating the hepatic phenotype of OTCD [36]. Briefly, for the mentioned work we reprogrammed patient‐derived skin fibroblasts from two subjects—designated OTCD_1M and OTCD_2F—who both were suffering from fatal OTCD. The male patient—subject OTCD_1M—died in the neonatal period due to hyperammonemia and multiorgan failure caused by a previously described fatal pathogenic variant in the *OTC* gene (c.548A>G [p.Tyr183Cys]) [47]. In our cellular model, OTCD_1M hiPSC‐Heps had absent OTC expression in line with the severe phenotype observed in this patient. The female patient—subject OTCD_2F—suffered from fatal hyperammonemia and liver failure at the age of 6 years due to a previously described stop mutation in the *OTC* gene (c.274C>T [p.Arg92*]) [48]. In OTCD_2F hiPSC‐Heps, no OTC protein expression or OTC activity was detectable, in line with the severe phenotype observed in this patient, which was caused by skewed XCI [36]. Since the generation and characterization of hiPSC‐Heps from both of these patients revealed absent OTC enzyme expression and activity [36], we hypothesized that these lines are not suitable or certainly not ideal candidate lines to study a potential interaction with PCs (Figure S1).

**FIGURE 4:**
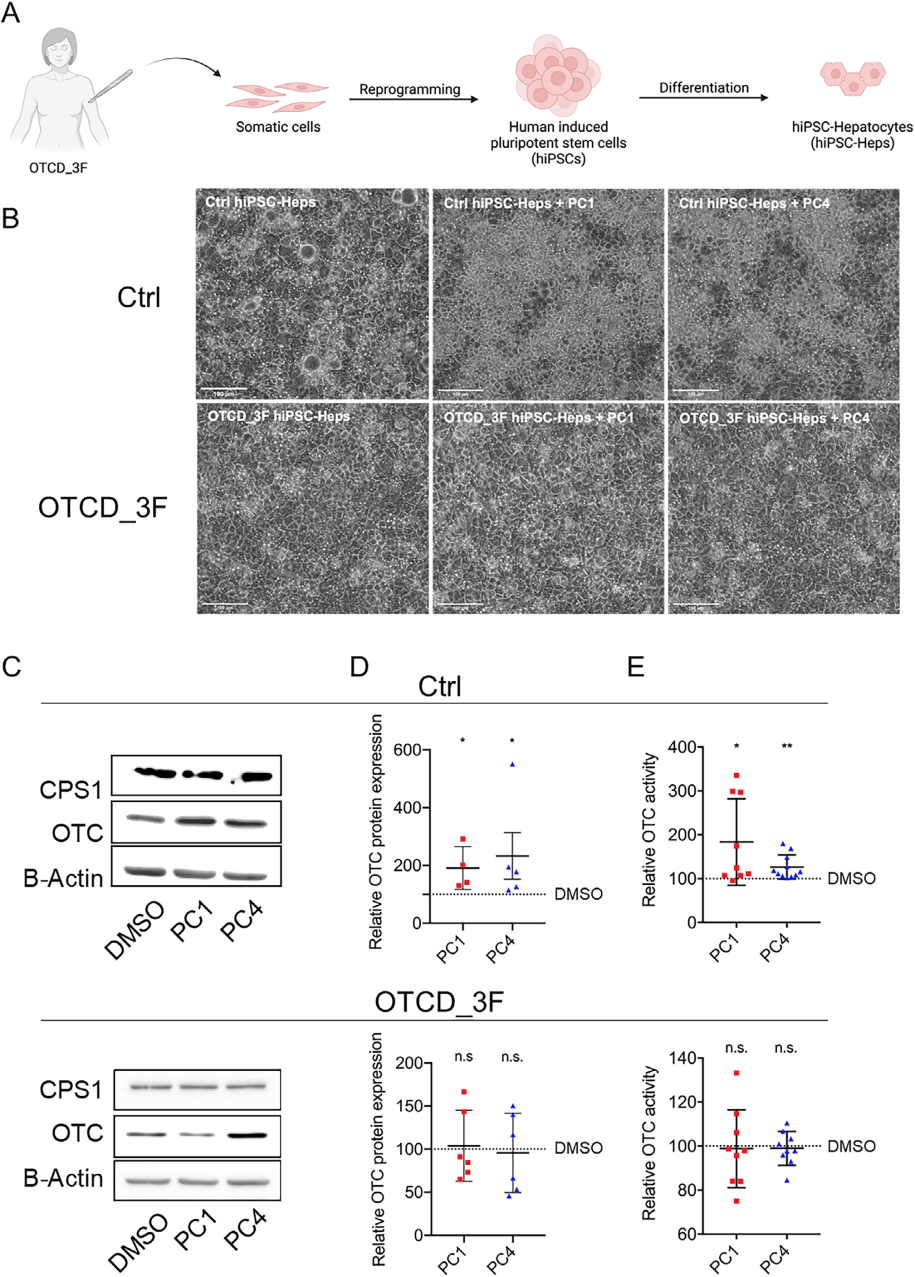
PC1 and PC4 enhance wild‐type OTC expression and activity in control hiPSC‐Heps. (A) Schematic representation of skin biopsy and fibroblast culture from OTCD_3F patient to generate human induced pluripotent stem cells (hiPSCs) differentiated into patient‐derived hiPSC‐hepatocytes (hiPSC‐Heps). Created with BioRender.com. (B) Brightfield microscopy showing representative images of control (Ctrl) and patient‐derived hiPSC‐Heps (OTCD_3F) treated as described in Figure 2A. Scale bar = 100 μM. (C and D) Representative western blot images of OTC and CPS1 (and B‐Actin as loading control) in Ctrl and patient‐derived hiPSC‐Heps (OTCD_3F) treated as described above and quantification of relative OTC expression. (E) Relative OTC activity in cells treated as described above. Data represent the average of 4–11 independent biological samples. One‐way ANOVA test; *p < 0.05, **p < 0.01. n.s., not significant. Error bars represent SEM.

Thus, for the present study, we generated a novel hiPSC‐Hep line from an additional female OTCD patient (OTCD_3F) who was diagnosed with OTCD at the age of 52 years when she suffered from a first reported metabolic decompensation with hyperammonemia, as published previously [49]. Molecular genetic analysis revealed a previously described pathogenic variant in the *OTC* gene (c.119G>A [p.Arg40His]). Patient‐derived fibroblasts were reprogrammed into hiPSCs, which were subsequently differentiated into hiPSC‐Heps according to the previously published protocol [36, 50] (Figure 4A).

### 3.5 PC1 and PC4 Enhance Wild‐Type OTC Protein Expression and Activity in hiPSC‐Heps

Control (Ctrl) and patient‐derived hiPSC‐Heps (OTCD_3F) were treated for 24 h with PC1 or PC4 and challenged with 1 mM ^15^NH_4_Cl as described above for PHHs (Figure 2A). Brightfield microscopy images revealed no differences in cell morphology after PC treatment, rendering relevant cytotoxicity unlikely (Figure 4B). The effect of PC1 and PC4 on OTC protein expression in Ctrl and OTCD_3F hiPSC‐Heps was assessed by western blot (Figure 4C) and was quantified relative to the B‐Actin expression (Figure 4D). CPS1—the enzyme catalyzing the entry of ammonia into the urea cycle—was used as a negative control for PC treatment, that is, to exclude unspecific changes in protein expression in response to PC treatment (Figure 4C). While PC1 and PC4 treatment revealed a significant increase in OTC protein expression in Ctrl hiPSC‐Heps (Figure 4C,D), this effect was not observed in OTCD_3F hiPSC‐Heps (Figure 4C,D). In line with the observed increase in OTC protein expression in Ctrl hiPSC‐Heps (Figure 4C,D), OTC activity also increased significantly in response to PC1 and PC4 treatment (Figure 4E). On the other hand, OTC activity in hiPSC‐Heps from OTCD_3F remained unchanged upon PC1 and PC4 treatment (Figure 4E), in line with the unaltered OTC protein expression (Figure 4C,D).

To conclude, PC1 and PC4 treatment revealed a positive effect on (wild‐type) OTC protein resulting in a significant upregulation of OTC protein expression in Ctrl hiPSC‐Heps (Figure 4C,D) and OTC activity (Figure 4E). This effect could not be replicated in patient‐derived OTCD_3F hiPSC‐Heps. These results suggest that PC1 and PC4 promote stabilization of wild‐type OTC protein, thereby enhancing OTC enzymatic activity.

### 3.6 PC4 Enhances OTC Expression and Activity in OTCD Patient‐Derived PHHs

**FIGURE 5:**
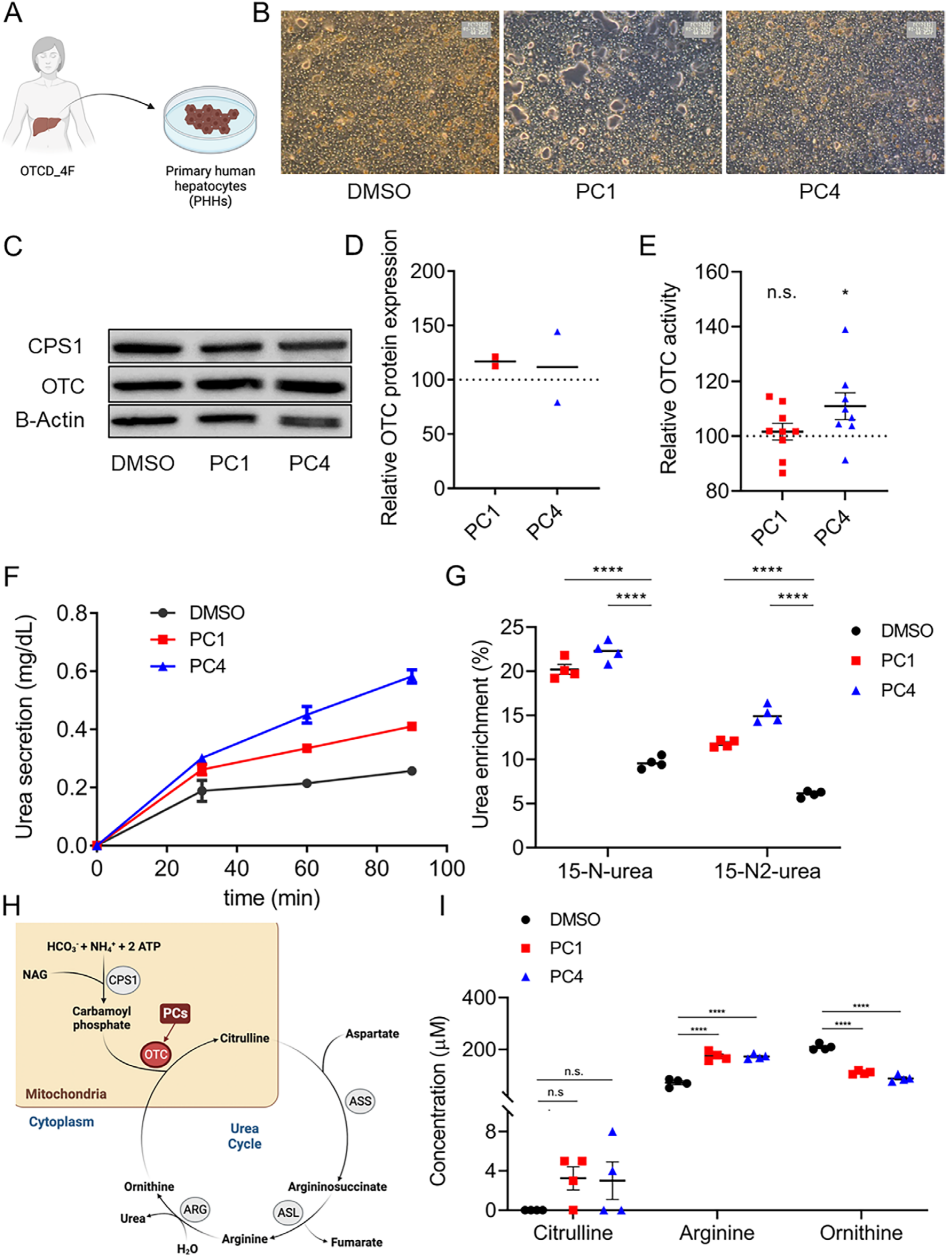
PC4 increases OTC activity and ureagenesis in OTC‐deficient patient‐derived PHHs. (A) Schematic representation of the origin of patient‐derived primary human hepatocytes (PHHs) isolated from liver explant of OTCD_4F patient. Created with BioRender.com. (B) Brightfield microscopy showing representative images of patient‐derived PHHs treated with PCs and ^15^NH_4_Cl as described in Figure 2A. (C–E) Western blot images of OTC and CPS1 (and B‐Actin as loading control) in cells treated as described above (C) and quantification of relative OTC expression (each two biological replicates available) (D) and relative OTC activity (E). (F and G) Urea secretion in PHHs in response to short‐term exposure (30, 60, and 90 min) with PC1 and PC4 compared to DMSO‐treated control cells (F) and ureagenesis assay showing ^15^N‐urea and ^15^N_2_‐urea enrichment in cells treated for 24 h, respectively (G). (H) Schematic showing urea cycle and selected relevant amino acids. Created with BioRender.com. (I) Quantification of selected amino acids citrulline, arginine, and ornithine in cell culture supernatants. Data represent the average of two (D; no statistical analysis performed) and four to nine (E–H) biological samples. Student's *t*‐test (E) or one‐way ANOVA test (H); *p < 0.05, ****p < 0.0001. n.s., not significant. Error bars represent SEM.

To further evaluate the effects of the previously tested PCs, we tested PC1 and PC4 on cultured PHHs, which were isolated from a liver explant derived from a female OTCD patient designated OTCD_4F (Figure 5A) [51]. In this female patient, OTCD was confirmed by severely reduced OTC enzyme activity in the patient's liver explant (residual OTC activity 17%).

Similarly to the previous experiments, OTCD_4F PHHs were pretreated for 24 h with PC1 and PC4 before concomitant exposure to ^15^NH_4_Cl (Figure 2A). Brightfield microscopy did not reveal significant cytotoxic effects (Figure 5B).

To determine if the PCs showed any stabilizing effect as previously observed for wild‐type OTC protein in Ctrl hiPSC‐Heps (Figure 4C,D), OTC protein expression (Figure 5C,D) and activity (Figure 5E) were assessed. While we did not have enough biological replicates to assess a detectable effect on OTC protein expression (Figure 5C,D), treatment with PC4 revealed a significant increase in OTC enzymatic activity compared to DMSO‐treated PHHs from OTCD_4F (Figure 5E). This effect was not observed when treating PHHs with PC1 (Figure 5E). Urea secretion and ureagenesis, both were significantly increased in response to PC1 and PC4 treatment after a concomitant challenge with ^15^NH_4_Cl of OTCD_4F PHHs (Figure 5F,G). Determination of amino acid profiles displayed differences in measured concentrations of the amino acids citrulline, arginine, and ornithine, all linked to the urea cycle (Figure 5H,I). Citrulline concentrations showed a non‐significant tendency to increase after PC1 and PC4 treatment compared to DMSO‐treated control OTCD_4F PHHs, indicating increased PC‐mediated enzymatic OTC catalysis (Figure 5I). Arginine increased significantly, and ornithine decreased significantly in response to PC1 and PC4 treatment, in line with the results above revealing increased PC‐mediated OTC activity and concomitant urea secretion and ureagenesis (Figure 5E,H,I).

Taken together, these results suggest a PC‐mediated increase in OTC activity and ureagenesis in OTCD_4F patient‐derived PHHs.

## 4 Discussion

OTCD is the most common and often fatal UCD [52] and is currently without curative treatment apart from liver transplantation. Therefore, there is an urgent need for novel treatment options. In the present work, we investigated a new treatment strategy by using PCs. The clinical application of PCs has been recently established as a potential treatment option for several inborn errors of metabolism as they have the ability to stabilize deficient enzymes [20].

To study the role of PCs as a potential novel treatment for OTCD, we first performed a DSF‐monitored HTS using more than 10 000 compounds with purified wild‐type OTC protein. Compounds that were able to thermostabilize OTC were selected as primary hits and underwent further stratification processes, yielding five potential PCs (PC1–PC5; Figure 1A,B) for evaluation in PHHs from healthy controls (Figure 2A–E). Subsequently, PC1 and PC4 were further tested in different patient‐derived liver disease models.

We previously developed the here applied liver disease model generating multiple patient‐derived hiPSC‐Heps of several OTC‐deficient patients [36, 51]. The considerable advantage of this model is that it enables performing drug screenings in a patient‐specific and highly personalized way. Further, such a model can be used as a diagnostic tool in OTC‐deficient patients lacking molecular genetic confirmation, as previously reported for patient OTCD_4F [51]. In addition, this hiPSC‐derived and OTCD patient‐derived liver disease model may be used to test standard drugs for OTCD, such as sodium phenylbutyrate. Last but not least, this model should allow researchers to investigate potential dietary regimens as recently reported for MDH2 deficiency (another inborn error of metabolism) [53]. Here, we applied this in vitro disease model and evaluated the effects of PC1 and PC4 in control and patient‐derived hiPSC‐Heps from a female OTCD patient (designated OTCD_3F). While PC1 and PC4 stabilized wild‐type OTC protein and enhanced OTC activity in hiPSC‐Heps from a healthy control, these effects were not observed in hiPSC‐Heps from the here investigated OTCD_3F patient (Figure 4A–E). As previously reported, in OTCD_1M and OTCD_2F, there was a complete lack of OTC protein expression [36], and hence, PCs could expectedly not interact with OTC to exert a positive effect (Figure S1A,B). The proposed mechanism of action for PC1 and PC4 is a direct interaction of both PCs with the OTC protein, thereby enhancing OTC stability and activity. Generally, hiPSC‐Hep lines not expressing OTC protein—for example, due to an early stop mutation causing nonsense‐mediated mRNA decay—are probably not suitable patient lines to treat with PCs.

Additional lines harboring pathogenic *OTC* variants causing misfolding of OTC should be evaluated for a PC‐mediated positive effect. Moreover, pathogenic *OTC* variants expressing reduced wild‐type OTC enzyme, for example, due to aberrant mRNA splicing [54, 55] could be particularly promising candidates since they express a variable amount of wild‐type enzyme.

Our developed OTCD liver disease model using patient‐derived hiPSC‐Heps would be specifically suitable for this approach.

As recently reported by our group, studying hiPSC‐Heps in the context of UCDs brings along some major challenges since these cells do not have a complete functional ureagenesis capacity [36].

Thus, for this work, we took the unique opportunity to evaluate the effects of PC1 and PC4 in patient‐derived PHHs received from a liver explant from another female OTCD patient (designated OTCD_4F) who underwent liver transplantation due to recurrent metabolic decompensations (Figure 5A–I) [51]. In these patient‐derived PHHs—still considered the gold standard to perform liver‐related in vitro studies—PC4 enhanced OTC expression and activity as well as ureagenesis, implying a stabilizing effect of PC4 on (wild‐type) OTC protein. Increasing OTC activity only by a few percent—for example, restoring the residual enzyme activity from 5% to 10%—might be therapeutic in heterozygous female OTCD patients [56]. Thus, females with unfavorable/skewed XCI causing predominant expression of the mutant OTC allele and only little of the wild‐type OTC protein might profit from PC4 treatment as it rescues OTC activity [57].

Some of the observed dissimilarities in this study are likely attributed to differences in the XCI pattern. While PC4 significantly enhanced OTC activity as well as ureagenesis in OTCD_4F PHHs, similar effects were not seen in hiPSC‐Heps from OTCD_3F—another female patient with late‐onset OTCD probably due to unfavorable/skewed XCI.

Based on previous experiments with PCs, an incubation time of 24 h and a concentration of 100 μM were considered reasonable. Further experiments to optimize these parameters as well as to verify whether these compounds are solely binding to OTC or whether there are off‐target effects, that is, compounds are binding and affecting the stability and activity of other proteins, would be the next required step before bringing these compounds into clinical use. Further, a combination of the two compounds PC1 and PC4 might be a suitable approach.

In summary, in this work, we identified two PCs—PC1 and PC4—that increase OTC protein stability and enhance enzymatic activity, revealing their potential as a new treatment strategy for OTCD. This method could be improved by performing an HTS on specific patient‐derived pathogenic *OTC* variants to find appropriate and specific stabilizing PCs for each affected individual. Further experiments in patient‐derived hiPSC‐Heps from additional patients are required to systematically advance this model and to evaluate the utility of PCs as a potential and novel treatment strategy for OTCD.

## Author Contributions

A.R. and M.B. assisted and/or performed experiments involving hiPSC‐Heps. A.R. generated respective figures and a preliminary draft of specific parts of the manuscript. J.U. and A.M. performed the DSF‐based HTS with purified OTC and gave input and contributed to this specific part of the manuscript and to the respective figures. D.M. contributed to the determination of the amino acids. S.M. provided us with the OTCD_3F hiPSC line, which he had previously generated. A.K. and V.B. were both involved in the experiments using PHHs of OTCD_4F. A.V.P. performed the computational modeling and made the respective figures. V.R. and N.G. generated purified human OTC protein, which was used in the HTS and checked the structural docking models for lack of structure‐based interference with trimer formation and with binding of bisubstrate or l‐ornithine analogs. V.R. provided critical review of the manuscript. G.A. and M.P. both developed the ureagenesis assay. B.T., J.H., and A.M. concepted the project part containing the HTS and provided critical review of the manuscript. A.L. concepted the overall study, designed and performed all experiments involving cells, made the figures, and wrote the manuscript. All the authors have read the manuscript and approved it.

## Conflicts of Interest

The authors declare no conflicts of interest.

## Supporting information


**Data S1** Supporting Information.

**Table S1.** Table.
