# Supplementary material for: Novel Treatment Strategy for Patients With Urea Cycle Disorders: Pharmacological Chaperones Enhance Enzyme Stability and Activity in Patient‐Derived Liver Disease Models
Source: J Inherit Metab Dis. 2025 May 27;48(3):e70043. doi: 10.1002/jimd.70043 (PMC12107682; doi:10.1002/jimd.70043)
Supplement: Supplementary file 1 — Data S1 Supporting Information. [file JIMD-48-0-s001.docx]

**SUPPORTING FIGURES AND TABLES**

**Novel treatment strategy for patients with urea cycle disorders: pharmacological chaperones enhance enzyme stability and activity in patient-derived liver disease models**

Adhuresa Ramosaj, Mariia Borsuk, Jarl Underhaug, Déborah Mathis, Shirou Matsumoto, Adrian Keogh, Vanessa Banz, Amit V Pandey, Nadine Gougeard, Vicente Rubio, Aurora Martinez, Gabriella Allegri, Martin Poms, Beat Thöny, Johannes Häberle, Alexander Laemmle

**SUPPORTING FIGURES**

A


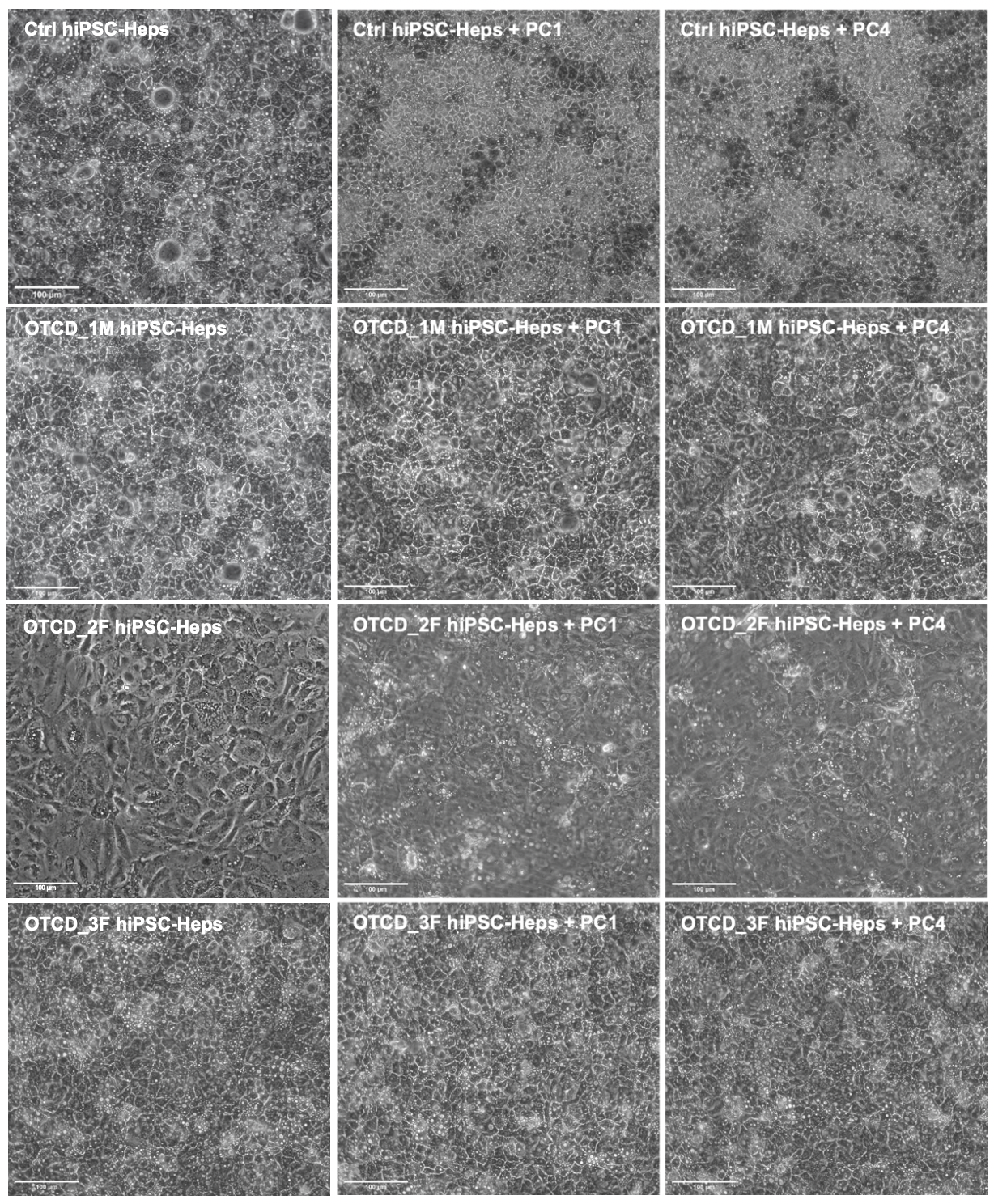


B


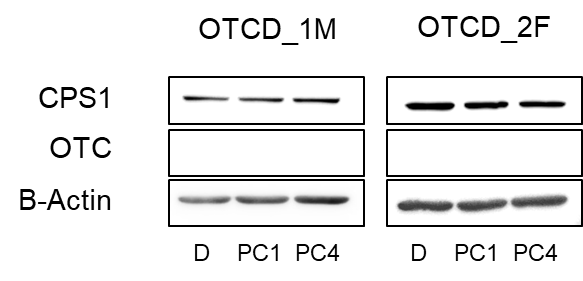


**Supporting Figure 1: PC1 and PC4 revealed no effect on OTC expression and activity in OTCD_1 and OTCD_2 hiPSC-Heps**

(A) Brightfield microscopy showing representative images of patient-derived hiPSC-Heps (OTCD_1M and OTCD_2F) treated as described in Figure 2A. Scale bar = 100 μM. (B) Representative western blot images of OTC and CPS1 (and B-Actin as loading control) in patient-derived hiPSC-Heps (OTCD_1M and OTCD_2F) treated as described above.

**SUPPORTING TABLES**

**Supporting Table S1 (see separate Excel-file)**

The cutoff value for hit selection was set to ΔTm ±1 °C, which corresponds to 5xSD for a typical plate. Validation of compound hits was performed by concentration dependent DSF assays.

**Supporting Table S2**

Calculation of binding of PC1 and PC4 to OTC protein. 25 VINA docking runs of the PC1 and PC4 bound to the OTC were analyzed, rescored and top binding poses were indicated. Positive energies indicate stronger binding. Calculations from two representative top poses are shown along with residues forming the binding pocket. Residues that are common across binding poses of both PC1 and PC4 are shown in bold and are likely to form a binding hotspot for the chaperone molecules.

|  | Binding energy  kCal/mol | Dissociation  constant µM | Contact residues |
| --- | --- | --- | --- |
| **PC1** |  |  |  |
| 1 | 6.6 | 13.7 | LYS 88 **ASP 165** LEU 166 GLY 197 ASN 198 ASN 199 **HIS 202** GLY 222 TYR 223 GLU 224 **ASP 226** SER 267 MET 268 GLN 270 **ARG 277** |
| 2 | 6.3 | 22.7 | LYS 88 TYR 143 **ASP 165** LEU 166 ASP 196 GLY 197 ASN 198 ASN 199 **HIS 202** TYR 223 PRO 225 **ASP 226** SER 267 MET 268 GLY 269 GLN 270 **ARG 277** |
| **PC4** |  |  |  |
| 1 | 5.7 | 59.9 | **ASP 165** LEU 166 ASP 196 GLY 197 ASN 198 ASN 199 **HIS 202** TYR 223 GLU 224 PRO 225 **ASP 226** SER 267 GLN 270 **ARG 277** |
| 2 | 5.4 | 102.5 | LYS 88 **ASP 165** LEU 166 ASP 196 GLY 197 ASN 198 ASN 199 **HIS 202** TYR 223 GLU 224 **ASP 226** SER 267 MET 268 GLY 269 GLN 270 **ARG 277** |
